# Crystal structure of 1′-ethyl­spiro[chroman-4,4′-imidazolidine]-2′,5′-dione: a hydantoine derivative

**DOI:** 10.1107/S2056989015016175

**Published:** 2015-09-12

**Authors:** S. B. Benaka Prasad, S. Naveen, M. Madaiah, N. K. Lokanath, Ismail Warad, Muneer Abdoh

**Affiliations:** a Department of Chemistry, School of Engineering and Technology, Jain University, Bangalore 562 112, India; b Institution of Excellence, University of Mysore, Manasagangotri, Mysuru 570 006, India; c Department of Studies in Chemistry, University of Mysore, Manasagangotri, Mysuru 570 006, India; d Department of Studies in Physics, University of Mysore, Manasagangotri, Mysuru 570 006, India; e Department of Chemistry, Science College, An-Najah National University, PO Box 7, Nablus, Palestinian Territories; f Department of Physics, Science College, An-Najah National University, PO Box 7, Nablus, Palestinian Territories

**Keywords:** crystal structure, hydantoin derivatives, imidazolidine, chroman, spiro, hydrogen bonding, C—H⋯π inter­actions

## Abstract

The title compound, C_13_H_13_N_2_O_3_, a hydantoin derivative, crystallized with two mol­ecules (*A* and *B*) in an asymmetric unit. In mol­ecule *A*, the imidazolidine ring is twisted about the C—N bond involving the spiro C atom, while in mol­ecule *B* this ring is flat (r.m.s. deviation = 0.010 Å). The pyran rings in both mol­ecules have distorted half-chair conformations. The mean plane of the imidazolidine ring is inclined to the aromatic ring of the chroman unit by 79.71 (11)° in mol­ecule *A* and 82.83 (12)° in mol­ecule *B*. In the crystal, pairs of N—H⋯O hydrogen bonds link the individual mol­ecules to form *A*–*A* and *B*–*B* inversion dimers. The dimers are linked *via* N—H⋯O and C—H⋯O hydrogen bonds, forming sheets lying parallel to the *bc* plane, *viz.* (011). Within the sheets, the *A* and *B* mol­ecules are linked by C—H⋯π inter­actions.

## Related literature   

For related literature on hydantoin derivatives, see: Manjunath *et al.* (2011 ▸, 2012 ▸).
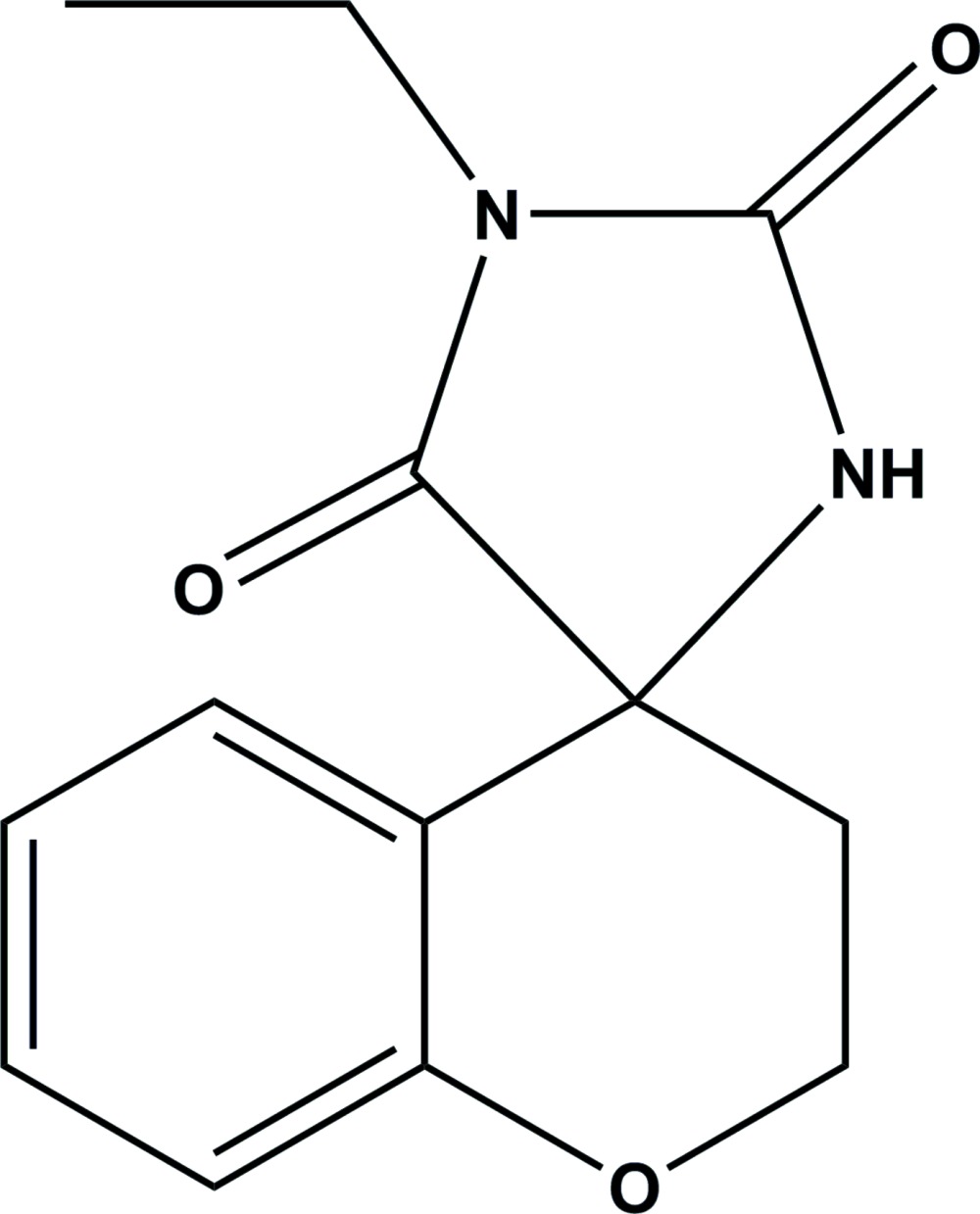


## Experimental   

### Crystal data  

C_13_H_14_N_2_O_3_
*M* *_r_* = 246.26
Triclinic, 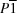

*a* = 10.2314 (16) Å
*b* = 11.0693 (18) Å
*c* = 11.3254 (19) Å
α = 91.736 (8)°
β = 98.695 (8)°
γ = 105.345 (8)°
*V* = 1219.4 (3) Å^3^
*Z* = 4
Cu *K*α radiation
μ = 0.80 mm^−1^
*T* = 296 K
0.23 × 0.22 × 0.21 mm

### Data collection  

Bruker X8 Proteum diffractometer
Absorption correction: multi-scan (*SADABS*; Bruker, 2013 ▸) *T*_min_ = 0.838, *T*_max_ = 0.850
14480 measured reflections
3990 independent reflections
3195 reflections with *I* > 2σ(*I*)
*R*_int_ = 0.054

### Refinement  

*R*[*F* ^2^ > 2σ(*F* ^2^)] = 0.064
*wR*(*F* ^2^) = 0.185
*S* = 1.04
3990 reflections
328 parameters
H-atom parameters constrained
Δρ_max_ = 0.36 e Å^−3^
Δρ_min_ = −0.39 e Å^−3^

### 

Data collection: *APEX2* (Bruker, 2013 ▸); cell refinement: *SAINT* (Bruker, 2013 ▸); data reduction: *SAINT*; program(s) used to solve structure: *SHELXS97* (Sheldrick, 2008 ▸); program(s) used to refine structure: *SHELXL97* (Sheldrick, 2008 ▸); molecular graphics: *Mercury* (Macrae *et al.*, 2008 ▸); software used to prepare material for publication: *SHELXL97* and *PLATON* (Spek, 2009 ▸).

## Supplementary Material

Crystal structure: contains datablock(s) global, I. DOI: 10.1107/S2056989015016175/su5194sup1.cif

Structure factors: contains datablock(s) n6. DOI: 10.1107/S2056989015016175/su5194Isup2.hkl

Supporting information file. DOI: 10.1107/S2056989015016175/su5194Isup3.cml

**Fig. 1.**
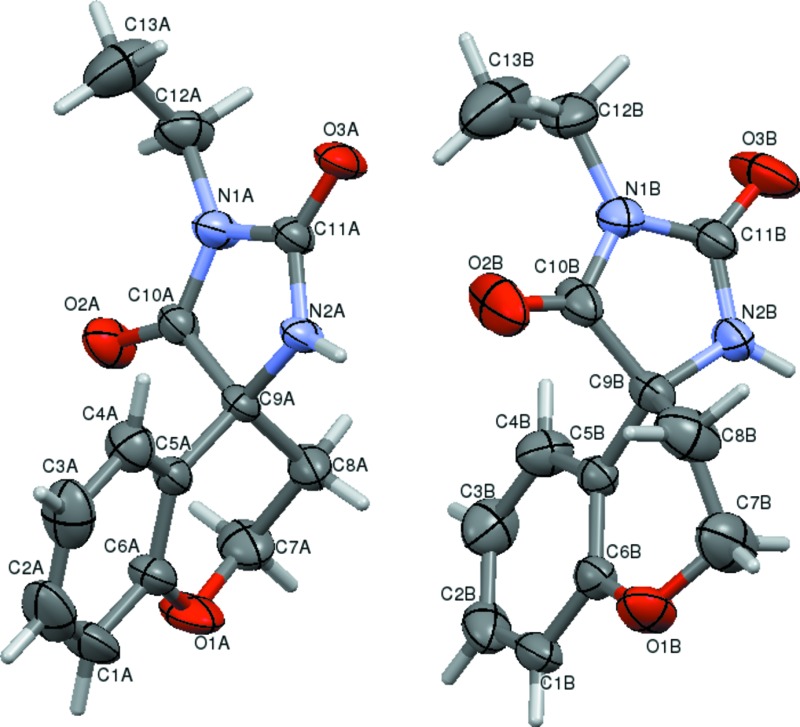
A view of the molecular structure of the two independent molecules of the title compound, with atom labelling. Displacement ellipsoids are drawn at the 50% probability level.

**Fig. 2.**
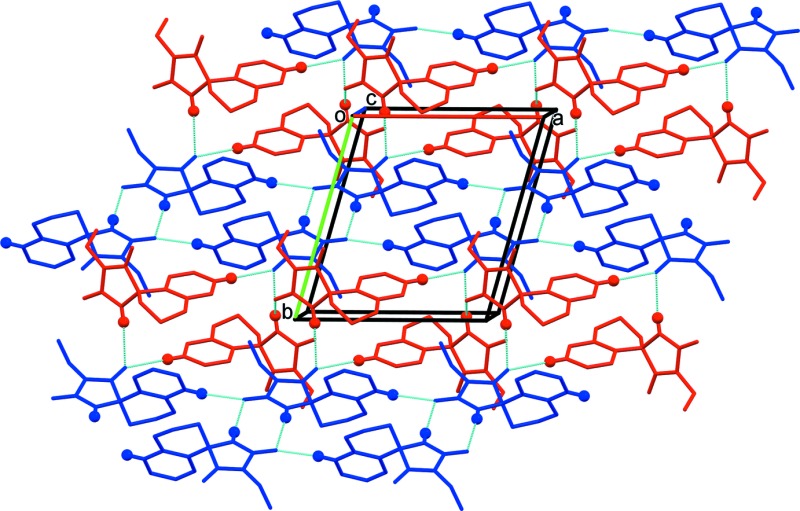
A viewed along the c axis of the crystal packing of the title compound (molecule A blue, molecule B red). The dashed lines represent hydrogen bonds (see Table 1; H atoms are shown as blue and red balls).

. DOI: 10.1107/S2056989015016175/su5194fig1.tif
A view of the mol­ecular structure of the two independent mol­ecules of the title compound, with atom labelling. Displacement ellipsoids are drawn at the 50% probability level.

c A B . DOI: 10.1107/S2056989015016175/su5194fig2.tif
A viewed along the *c* axis of the crystal packing of the title compound (mol­ecule *A* blue, mol­ecule *B* red). The dashed lines represent hydrogen bonds (see Table 1; H atoms are shown as blue and red balls).

CCDC reference: 1421223

Additional supporting information:  crystallographic information; 3D view; checkCIF report

## Figures and Tables

**Table 1:** Hydrogen-bond geometry (, ) *Cg* is the centroid of ring C1*A*C6*A*.

*D*H*A*	*D*H	H*A*	*D* *A*	*D*H*A*
N2*A*H2*A*O3*A* ^i^	0.86	2.06	2.857(3)	155
N2*B*H2*B*1O3*B* ^ii^	0.86	2.44	3.019(3)	124
N2*B*H2*B*1O2*A* ^iii^	0.86	2.55	3.290(3)	145
C1*A*H1*A*O3*A* ^iv^	0.93	2.45	3.263(4)	146
C2*B*H2*B*O2*A* ^v^	0.93	2.58	3.501(4)	173
C7*B*H7*B*2*Cg* ^iii^	0.93	2.99	3.680(3)	129

Symmetry codes: (i) 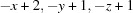; (ii) 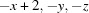; (iii) 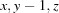; (iv) 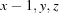; (v) 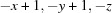.

## supplementary crystallographic information

### S1. Comment

Hydantoins are important precursors in the organic synthesis of natural and non-natural amino acids, *via* acid-, base- or enzyme-catalyzed hydrolysis. The hydantoin nucleus, containing an active urea moiety, is well known for its diverse biological activities such as lowering blood sugar levels in mammals, and anti-inflammatory and anti-microbial activity. Considerable inter­est has been shown towards the synthesis and characterization of hydantoin derivatives which is a novel class of heterocyclic compounds. As a part of our ongoing research on hydantoins (Manjunath *et al.*, 2011, 2012), the synthesis, characterization and the structural work of the title compound was undertaken and herein we report on its crystal structure.

The title compound, Fig. 1, an hydantoin derivative, crystallized with two molecules (A and B) in an asymmetric unit. In molecule A the imidazolidine ring is twisted about the C9A—N2A bond, while in molecule B this ring is flat (r.m.s. deviation = 0.010 Å). The pyran rings of the chroman units in both molecules have distorted half-chair conformations. The mean plane of the imidazolidine ring is inclined to the aromatic ring of the chroman unit by 79.71 (11) ° in molecule A and 82.83 (12) ° in molecule B.

In the crystal, pairs of N—H···O hydrogen bonds link the individual molecules to form A–A and B–B inversion dimers (Fig. 2 and Table 1). The dimers are linked via N—H···O and C—H···O hydrogen bonds forming sheets lying parallel to the bc plane, viz. (011); see Fig. 2 and Table 1. Within the sheets the A and B molecules are linked by C—H···π inter­actions (Table 1).

### S2. Synthesis and crystallization

A solution of 3-ethyl-5-(isochromon) imidazolidine-2, 4-dione (1.0 eq) in *N*,*N*-di­methyl formamide was taken, anhydrous K_2_CO_3_ (3.0 eq) was added to the solution and stirred for 10 min. 1-bromo-ethane (1–1.1eq) was added. The reaction mixture was stirred at room temperature for 8 h and the progress monitored by TLC. Upon completion, the solvent was removed under reduced pressure and the residue was taken in water and extracted with ethyl acetate. The organic was washed with water and then and dried over anhydrous sodium sulfate. The solvent was evaporated and the crude product was purified by column chromatography using chloro­form: methanol (9:1) as eluent. Single crystals were obtained by slow evaporation of a solution of the title compound in ethyl­acetate (M.p.: 572.1 K). Spectroscopic data: H^1^NMR (DMSO, 400 MHz) δ: 8.9 (s, 1H, NH), δ: 6.9(m, 3H, Ar—H) δ: 7.3 (m, 1H, Ar—H) δ: 4.5(m, 2H, CH_2_) δ: 2.5(m, 2H, CH_2_) δ: 2.3(m, 2H, CH_2_) δ: 1.1(m, 3H, CH_3_).

### S3. Refinement

Crystal data, data collection and structure refinement details are summarized in Table 2. The hydrogen atoms were fixed geometrically (N—H = 0.86 Å, C—H = 0.93–0.96 Å) and allowed to ride on their parent atoms with *U*_iso_(H) = 1.5*U*_eq_(*C*-methyl) and 1.2*U*_eq_(N,C) for other H atoms.

### Crystal data

**Table:**

C_13_H_14_N_2_O_3_	*Z* = 4
*M**_r_* = 246.26	*F*(000) = 520
Triclinic, *P*1	*D*_x_ = 1.341 Mg m^−^^3^
Hall symbol: -P 1	Cu *K*α radiation, λ = 1.54178 Å
*a* = 10.2314 (16) Å	Cell parameters from 3990 reflections
*b* = 11.0693 (18) Å	θ = 4.0–64.6°
*c* = 11.3254 (19) Å	µ = 0.80 mm^−^^1^
α = 91.736 (8)°	*T* = 296 K
β = 98.695 (8)°	Rectangle, green
γ = 105.345 (8)°	0.23 × 0.22 × 0.21 mm
*V* = 1219.4 (3) Å^3^	

### Data collection

**Table:**

Bruker X8 Proteum diffractometer	3990 independent reflections
Radiation source: Bruker MicroStar microfocus rotating anode	3195 reflections with *I* > 2σ(*I*)
Helios multilayer optics monochromator	*R*_int_ = 0.054
Detector resolution: 18.4 pixels mm^-1^	θ_max_ = 64.6°, θ_min_ = 4.0°
φ and ω scans	*h* = −11→11
Absorption correction: multi-scan (*SADABS*; Bruker, 2013)	*k* = −12→12
*T*_min_ = 0.838, *T*_max_ = 0.850	*l* = −13→13
14480 measured reflections	

### Refinement

**Table:**

Refinement on *F*^2^	Secondary atom site location: difference Fourier map
Least-squares matrix: full	Hydrogen site location: inferred from neighbouring sites
*R*[*F*^2^ > 2σ(*F*^2^)] = 0.064	H-atom parameters constrained
*wR*(*F*^2^) = 0.185	*w* = 1/[σ^2^(*F*_o_^2^) + (0.1316*P*)^2^ + 0.1551*P*] where *P* = (*F*_o_^2^ + 2*F*_c_^2^)/3
*S* = 1.04	(Δ/σ)_max_ < 0.001
3990 reflections	Δρ_max_ = 0.36 e Å^−^^3^
328 parameters	Δρ_min_ = −0.39 e Å^−^^3^
0 restraints	Extinction correction: *SHELXL97* (Sheldrick, 2008), Fc^*^=kFc[1+0.001xFc^2^λ^3^/sin(2θ)]^-1/4^
Primary atom site location: structure-invariant direct methods	Extinction coefficient: 0.025 (2)

### Special details

**Table:**

Geometry. All esds (except the esd in the dihedral angle between two l.s. planes) are estimated using the full covariance matrix. The cell esds are taken into account individually in the estimation of esds in distances, angles and torsion angles; correlations between esds in cell parameters are only used when they are defined by crystal symmetry. An approximate (isotropic) treatment of cell esds is used for estimating esds involving l.s. planes.
Refinement. Refinement on *F*^2^ for ALL reflections except those flagged by the user for potential systematic errors. Weighted *R*-factors *wR* and all goodnesses of fit *S* are based on *F*^2^, conventional *R*-factors *R* are based on *F*, with *F* set to zero for negative *F*^2^. The observed criterion of *F*^2^ > σ(*F*^2^) is used only for calculating -*R*-factor-obs *etc*. and is not relevant to the choice of reflections for refinement. *R*-factors based on *F*^2^ are statistically about twice as large as those based on *F*, and *R*-factors based on ALL data will be even larger.

### Fractional atomic coordinates and isotropic or equivalent isotropic displacement parameters (Å^2^)

**Table:**

	*x*	*y*	*z*	*U*_iso_*/*U*_eq_	
O1B	0.57043 (18)	0.04669 (17)	0.27294 (15)	0.0534 (5)	
N2A	0.88853 (16)	0.57988 (16)	0.41739 (15)	0.0350 (4)	
H2A	0.8777	0.5352	0.4778	0.042*	
O3A	1.12385 (15)	0.62164 (16)	0.42909 (15)	0.0458 (4)	
O2A	0.80600 (17)	0.76449 (16)	0.18917 (14)	0.0492 (5)	
C9B	0.8442 (2)	0.1368 (2)	0.20830 (18)	0.0377 (5)	
N1B	1.06259 (18)	0.25381 (17)	0.18235 (17)	0.0421 (5)	
O3B	1.12728 (19)	0.1008 (2)	0.08118 (19)	0.0704 (6)	
O2B	0.9417 (2)	0.35827 (19)	0.2803 (2)	0.0767 (7)	
C2B	0.4752 (3)	0.1850 (2)	0.0004 (2)	0.0532 (6)	
H2B	0.3962	0.1978	−0.0437	0.064*	
C1B	0.4685 (2)	0.1271 (2)	0.1051 (2)	0.0461 (6)	
H1B	0.3851	0.1010	0.1327	0.055*	
C6B	0.5869 (2)	0.10716 (19)	0.17046 (19)	0.0364 (5)	
C5B	0.7129 (2)	0.14862 (18)	0.13279 (17)	0.0326 (5)	
C10B	0.9517 (2)	0.2642 (2)	0.2298 (2)	0.0428 (6)	
C12B	1.1849 (3)	0.3580 (3)	0.1783 (3)	0.0597 (7)	
H12A	1.2625	0.3246	0.1728	0.072*	
H12B	1.2061	0.4116	0.2519	0.072*	
C13B	1.1641 (4)	0.4335 (3)	0.0750 (3)	0.0881 (11)	
H13A	1.1406	0.3802	0.0022	0.132*	
H13B	1.2472	0.4982	0.0729	0.132*	
H13C	1.0911	0.4711	0.0829	0.132*	
C11B	1.0424 (2)	0.1332 (2)	0.1297 (2)	0.0418 (5)	
N2B	0.9179 (2)	0.06477 (17)	0.14631 (19)	0.0458 (5)	
H2B1	0.8847	−0.0135	0.1229	0.055*	
C7B	0.6800 (3)	−0.0049 (3)	0.3177 (3)	0.0671 (8)	
H7B1	0.6795	−0.0746	0.2636	0.081*	
H7B2	0.6665	−0.0367	0.3951	0.081*	
C8B	0.8153 (3)	0.0914 (3)	0.3303 (2)	0.0624 (8)	
H8B1	0.8875	0.0557	0.3662	0.075*	
H8B2	0.8150	0.1622	0.3829	0.075*	
C3B	0.5997 (3)	0.2246 (2)	−0.0401 (2)	0.0564 (7)	
H3B	0.6043	0.2632	−0.1117	0.068*	
C4B	0.7163 (3)	0.2068 (2)	0.0259 (2)	0.0472 (6)	
H4B	0.7996	0.2343	−0.0016	0.057*	
C2A	0.4997 (3)	0.7412 (3)	0.5220 (3)	0.0669 (9)	
H2A1	0.4398	0.7705	0.5619	0.080*	
C1A	0.4498 (3)	0.6678 (3)	0.4165 (3)	0.0624 (8)	
H1A	0.3566	0.6473	0.3852	0.075*	
C6A	0.5395 (2)	0.6243 (2)	0.3564 (2)	0.0426 (5)	
C5A	0.6788 (2)	0.65461 (17)	0.40196 (18)	0.0321 (5)	
C9A	0.77539 (19)	0.60575 (17)	0.33690 (17)	0.0301 (5)	
C10A	0.8545 (2)	0.70485 (19)	0.26353 (18)	0.0334 (5)	
N1A	0.98963 (18)	0.71123 (17)	0.29492 (16)	0.0379 (5)	
C12A	1.1017 (2)	0.7874 (2)	0.2399 (2)	0.0506 (6)	
H12C	1.0633	0.8149	0.1652	0.061*	
H12D	1.1604	0.7359	0.2214	0.061*	
C13A	1.1862 (4)	0.8994 (3)	0.3188 (3)	0.0823 (10)	
H13D	1.1278	0.9484	0.3410	0.123*	
H13E	1.2535	0.9495	0.2768	0.123*	
H13F	1.2317	0.8727	0.3896	0.123*	
C11A	1.0107 (2)	0.63310 (19)	0.38692 (18)	0.0338 (5)	
C8A	0.6919 (2)	0.4888 (2)	0.2553 (2)	0.0440 (5)	
H8A1	0.7491	0.4656	0.2027	0.053*	
H8A2	0.6625	0.4191	0.3039	0.053*	
C7A	0.5674 (3)	0.5145 (2)	0.1813 (2)	0.0536 (6)	
H7A1	0.5976	0.5809	0.1295	0.064*	
H7A2	0.5160	0.4396	0.1308	0.064*	
O1A	0.48007 (17)	0.55034 (18)	0.25370 (17)	0.0615 (5)	
C4A	0.7264 (3)	0.7287 (2)	0.5093 (2)	0.0455 (6)	
H4A	0.8194	0.7496	0.5413	0.055*	
C3A	0.6370 (4)	0.7718 (3)	0.5691 (3)	0.0632 (8)	
H3A	0.6699	0.8212	0.6408	0.076*	

### Atomic displacement parameters (Å^2^)

**Table:**

	*U*^11^	*U*^22^	*U*^33^	*U*^12^	*U*^13^	*U*^23^
O1B	0.0430 (10)	0.0677 (11)	0.0567 (10)	0.0183 (8)	0.0215 (8)	0.0207 (8)
N2A	0.0209 (9)	0.0421 (9)	0.0451 (10)	0.0126 (7)	0.0050 (7)	0.0174 (7)
O3A	0.0213 (8)	0.0583 (9)	0.0611 (10)	0.0156 (7)	0.0050 (6)	0.0213 (8)
O2A	0.0424 (10)	0.0625 (10)	0.0488 (9)	0.0236 (8)	0.0055 (7)	0.0259 (8)
C9B	0.0305 (12)	0.0451 (11)	0.0411 (11)	0.0170 (9)	0.0061 (9)	−0.0006 (9)
N1B	0.0270 (10)	0.0398 (9)	0.0558 (11)	0.0071 (8)	0.0002 (8)	−0.0040 (8)
O3B	0.0336 (11)	0.0931 (14)	0.0857 (14)	0.0227 (10)	0.0111 (9)	−0.0303 (11)
O2B	0.0564 (13)	0.0689 (12)	0.0997 (16)	0.0263 (10)	−0.0094 (10)	−0.0475 (11)
C2B	0.0434 (15)	0.0453 (13)	0.0651 (16)	0.0159 (11)	−0.0148 (11)	−0.0012 (11)
C1B	0.0276 (12)	0.0452 (12)	0.0655 (15)	0.0129 (9)	0.0036 (10)	−0.0038 (11)
C6B	0.0328 (12)	0.0355 (10)	0.0421 (12)	0.0106 (8)	0.0084 (9)	−0.0007 (8)
C5B	0.0298 (11)	0.0333 (10)	0.0357 (10)	0.0106 (8)	0.0058 (8)	−0.0015 (8)
C10B	0.0348 (13)	0.0447 (12)	0.0476 (12)	0.0170 (10)	−0.0053 (9)	−0.0139 (10)
C12B	0.0346 (15)	0.0528 (14)	0.0792 (18)	−0.0012 (11)	−0.0063 (12)	0.0069 (13)
C13B	0.074 (2)	0.075 (2)	0.094 (2)	−0.0069 (17)	−0.0054 (18)	0.0300 (18)
C11B	0.0268 (12)	0.0500 (12)	0.0499 (12)	0.0164 (9)	0.0025 (9)	−0.0078 (10)
N2B	0.0318 (11)	0.0342 (9)	0.0725 (13)	0.0116 (7)	0.0105 (9)	−0.0106 (9)
C7B	0.0629 (19)	0.0815 (19)	0.0674 (18)	0.0299 (16)	0.0187 (14)	0.0356 (15)
C8B	0.0511 (17)	0.092 (2)	0.0509 (15)	0.0309 (15)	0.0064 (12)	0.0217 (14)
C3B	0.0576 (17)	0.0580 (15)	0.0461 (14)	0.0104 (12)	−0.0067 (11)	0.0136 (11)
C4B	0.0402 (14)	0.0547 (13)	0.0423 (12)	0.0055 (10)	0.0054 (10)	0.0088 (10)
C2A	0.072 (2)	0.0721 (18)	0.080 (2)	0.0415 (16)	0.0448 (17)	0.0179 (16)
C1A	0.0340 (15)	0.0741 (18)	0.092 (2)	0.0266 (13)	0.0259 (13)	0.0158 (16)
C6A	0.0264 (12)	0.0478 (12)	0.0557 (13)	0.0132 (9)	0.0069 (9)	0.0077 (10)
C5A	0.0258 (11)	0.0324 (9)	0.0413 (11)	0.0114 (8)	0.0075 (8)	0.0094 (8)
C9A	0.0208 (10)	0.0326 (9)	0.0379 (10)	0.0104 (8)	0.0010 (8)	0.0066 (8)
C10A	0.0288 (11)	0.0376 (10)	0.0374 (10)	0.0148 (8)	0.0056 (8)	0.0069 (8)
N1A	0.0258 (10)	0.0475 (10)	0.0439 (10)	0.0124 (8)	0.0093 (7)	0.0179 (8)
C12A	0.0373 (14)	0.0639 (15)	0.0531 (14)	0.0111 (11)	0.0164 (10)	0.0229 (12)
C13A	0.073 (2)	0.0676 (18)	0.089 (2)	−0.0131 (16)	0.0168 (18)	0.0178 (17)
C11A	0.0250 (11)	0.0387 (10)	0.0405 (11)	0.0130 (8)	0.0051 (8)	0.0103 (8)
C8A	0.0361 (13)	0.0371 (11)	0.0572 (13)	0.0133 (9)	−0.0016 (10)	−0.0067 (10)
C7A	0.0405 (15)	0.0555 (14)	0.0571 (15)	0.0112 (11)	−0.0091 (11)	−0.0111 (11)
O1A	0.0232 (9)	0.0751 (12)	0.0782 (13)	0.0112 (8)	−0.0085 (8)	−0.0123 (10)
C4A	0.0445 (14)	0.0475 (12)	0.0451 (12)	0.0148 (10)	0.0053 (10)	0.0031 (10)
C3A	0.088 (2)	0.0586 (15)	0.0546 (15)	0.0319 (15)	0.0275 (15)	0.0039 (12)

### Geometric parameters (Å, º)

**Table:**

O1B—C6B	1.367 (3)	C8B—H8B1	0.9700
O1B—C7B	1.422 (3)	C8B—H8B2	0.9700
N2A—C11A	1.335 (3)	C3B—C4B	1.373 (4)
N2A—C9A	1.458 (2)	C3B—H3B	0.9300
N2A—H2A	0.8600	C4B—H4B	0.9300
O3A—C11A	1.224 (2)	C2A—C3A	1.373 (5)
O2A—C10A	1.213 (2)	C2A—C1A	1.373 (5)
C9B—N2B	1.463 (3)	C2A—H2A1	0.9300
C9B—C5B	1.518 (3)	C1A—C6A	1.393 (3)
C9B—C8B	1.528 (3)	C1A—H1A	0.9300
C9B—C10B	1.528 (3)	C6A—O1A	1.363 (3)
N1B—C10B	1.356 (3)	C6A—C5A	1.387 (3)
N1B—C11B	1.397 (3)	C5A—C4A	1.392 (3)
N1B—C12B	1.468 (3)	C5A—C9A	1.513 (3)
O3B—C11B	1.217 (3)	C9A—C10A	1.529 (3)
O2B—C10B	1.208 (3)	C9A—C8A	1.537 (3)
C2B—C1B	1.368 (4)	C10A—N1A	1.356 (3)
C2B—C3B	1.385 (4)	N1A—C11A	1.402 (3)
C2B—H2B	0.9300	N1A—C12A	1.467 (3)
C1B—C6B	1.394 (3)	C12A—C13A	1.489 (4)
C1B—H1B	0.9300	C12A—H12C	0.9700
C6B—C5B	1.386 (3)	C12A—H12D	0.9700
C5B—C4B	1.389 (3)	C13A—H13D	0.9600
C12B—C13B	1.480 (4)	C13A—H13E	0.9600
C12B—H12A	0.9700	C13A—H13F	0.9600
C12B—H12B	0.9700	C8A—C7A	1.512 (3)
C13B—H13A	0.9600	C8A—H8A1	0.9700
C13B—H13B	0.9600	C8A—H8A2	0.9700
C13B—H13C	0.9600	C7A—O1A	1.421 (3)
C11B—N2B	1.343 (3)	C7A—H7A1	0.9700
N2B—H2B1	0.8600	C7A—H7A2	0.9700
C7B—C8B	1.491 (4)	C4A—C3A	1.386 (4)
C7B—H7B1	0.9700	C4A—H4A	0.9300
C7B—H7B2	0.9700	C3A—H3A	0.9300
			
C6B—O1B—C7B	114.36 (18)	C3B—C4B—C5B	121.6 (2)
C11A—N2A—C9A	112.58 (16)	C3B—C4B—H4B	119.2
C11A—N2A—H2A	123.7	C5B—C4B—H4B	119.2
C9A—N2A—H2A	123.7	C3A—C2A—C1A	120.7 (2)
N2B—C9B—C5B	113.58 (17)	C3A—C2A—H2A1	119.7
N2B—C9B—C8B	114.36 (19)	C1A—C2A—H2A1	119.7
C5B—C9B—C8B	110.01 (18)	C2A—C1A—C6A	119.7 (3)
N2B—C9B—C10B	100.06 (16)	C2A—C1A—H1A	120.2
C5B—C9B—C10B	110.45 (17)	C6A—C1A—H1A	120.2
C8B—C9B—C10B	107.8 (2)	O1A—C6A—C5A	123.96 (19)
C10B—N1B—C11B	111.56 (19)	O1A—C6A—C1A	115.4 (2)
C10B—N1B—C12B	124.7 (2)	C5A—C6A—C1A	120.7 (2)
C11B—N1B—C12B	123.6 (2)	C6A—C5A—C4A	118.42 (19)
C1B—C2B—C3B	120.0 (2)	C6A—C5A—C9A	120.34 (19)
C1B—C2B—H2B	120.0	C4A—C5A—C9A	121.23 (18)
C3B—C2B—H2B	120.0	N2A—C9A—C5A	113.19 (16)
C2B—C1B—C6B	119.9 (2)	N2A—C9A—C10A	100.66 (15)
C2B—C1B—H1B	120.1	C5A—C9A—C10A	112.17 (15)
C6B—C1B—H1B	120.1	N2A—C9A—C8A	111.52 (16)
O1B—C6B—C5B	122.99 (19)	C5A—C9A—C8A	108.84 (17)
O1B—C6B—C1B	116.03 (19)	C10A—C9A—C8A	110.28 (17)
C5B—C6B—C1B	121.0 (2)	O2A—C10A—N1A	126.28 (19)
C6B—C5B—C4B	117.72 (19)	O2A—C10A—C9A	126.74 (19)
C6B—C5B—C9B	121.36 (18)	N1A—C10A—C9A	106.96 (16)
C4B—C5B—C9B	120.86 (19)	C10A—N1A—C11A	111.53 (16)
O2B—C10B—N1B	125.3 (2)	C10A—N1A—C12A	125.46 (17)
O2B—C10B—C9B	126.8 (2)	C11A—N1A—C12A	123.00 (17)
N1B—C10B—C9B	107.88 (17)	N1A—C12A—C13A	112.6 (2)
N1B—C12B—C13B	111.7 (2)	N1A—C12A—H12C	109.1
N1B—C12B—H12A	109.3	C13A—C12A—H12C	109.1
C13B—C12B—H12A	109.3	N1A—C12A—H12D	109.1
N1B—C12B—H12B	109.3	C13A—C12A—H12D	109.1
C13B—C12B—H12B	109.3	H12C—C12A—H12D	107.8
H12A—C12B—H12B	107.9	C12A—C13A—H13D	109.5
C12B—C13B—H13A	109.5	C12A—C13A—H13E	109.5
C12B—C13B—H13B	109.5	H13D—C13A—H13E	109.5
H13A—C13B—H13B	109.5	C12A—C13A—H13F	109.5
C12B—C13B—H13C	109.5	H13D—C13A—H13F	109.5
H13A—C13B—H13C	109.5	H13E—C13A—H13F	109.5
H13B—C13B—H13C	109.5	O3A—C11A—N2A	129.04 (18)
O3B—C11B—N2B	128.9 (2)	O3A—C11A—N1A	123.54 (18)
O3B—C11B—N1B	123.8 (2)	N2A—C11A—N1A	107.40 (16)
N2B—C11B—N1B	107.23 (17)	C7A—C8A—C9A	110.44 (17)
C11B—N2B—C9B	113.24 (17)	C7A—C8A—H8A1	109.6
C11B—N2B—H2B1	123.4	C9A—C8A—H8A1	109.6
C9B—N2B—H2B1	123.4	C7A—C8A—H8A2	109.6
O1B—C7B—C8B	111.1 (2)	C9A—C8A—H8A2	109.6
O1B—C7B—H7B1	109.4	H8A1—C8A—H8A2	108.1
C8B—C7B—H7B1	109.4	O1A—C7A—C8A	112.2 (2)
O1B—C7B—H7B2	109.4	O1A—C7A—H7A1	109.2
C8B—C7B—H7B2	109.4	C8A—C7A—H7A1	109.2
H7B1—C7B—H7B2	108.0	O1A—C7A—H7A2	109.2
C7B—C8B—C9B	110.7 (2)	C8A—C7A—H7A2	109.2
C7B—C8B—H8B1	109.5	H7A1—C7A—H7A2	107.9
C9B—C8B—H8B1	109.5	C6A—O1A—C7A	118.11 (17)
C7B—C8B—H8B2	109.5	C3A—C4A—C5A	120.9 (2)
C9B—C8B—H8B2	109.5	C3A—C4A—H4A	119.6
H8B1—C8B—H8B2	108.1	C5A—C4A—H4A	119.6
C4B—C3B—C2B	119.8 (2)	C2A—C3A—C4A	119.7 (3)
C4B—C3B—H3B	120.1	C2A—C3A—H3A	120.2
C2B—C3B—H3B	120.1	C4A—C3A—H3A	120.2
			
C3B—C2B—C1B—C6B	−0.5 (3)	C3A—C2A—C1A—C6A	−0.2 (4)
C7B—O1B—C6B—C5B	−18.6 (3)	C2A—C1A—C6A—O1A	178.9 (2)
C7B—O1B—C6B—C1B	162.2 (2)	C2A—C1A—C6A—C5A	−0.2 (4)
C2B—C1B—C6B—O1B	−178.9 (2)	O1A—C6A—C5A—C4A	−178.5 (2)
C2B—C1B—C6B—C5B	1.9 (3)	C1A—C6A—C5A—C4A	0.5 (3)
O1B—C6B—C5B—C4B	178.82 (19)	O1A—C6A—C5A—C9A	0.3 (3)
C1B—C6B—C5B—C4B	−2.1 (3)	C1A—C6A—C5A—C9A	179.3 (2)
O1B—C6B—C5B—C9B	−4.1 (3)	C11A—N2A—C9A—C5A	−129.41 (18)
C1B—C6B—C5B—C9B	175.04 (18)	C11A—N2A—C9A—C10A	−9.5 (2)
N2B—C9B—C5B—C6B	122.3 (2)	C11A—N2A—C9A—C8A	107.5 (2)
C8B—C9B—C5B—C6B	−7.3 (3)	C6A—C5A—C9A—N2A	−146.34 (19)
C10B—C9B—C5B—C6B	−126.2 (2)	C4A—C5A—C9A—N2A	32.5 (3)
N2B—C9B—C5B—C4B	−60.6 (3)	C6A—C5A—C9A—C10A	100.6 (2)
C8B—C9B—C5B—C4B	169.7 (2)	C4A—C5A—C9A—C10A	−80.6 (2)
C10B—C9B—C5B—C4B	50.9 (2)	C6A—C5A—C9A—C8A	−21.7 (2)
C11B—N1B—C10B—O2B	−179.7 (2)	C4A—C5A—C9A—C8A	157.10 (19)
C12B—N1B—C10B—O2B	−4.0 (4)	N2A—C9A—C10A—O2A	−173.9 (2)
C11B—N1B—C10B—C9B	0.5 (2)	C5A—C9A—C10A—O2A	−53.3 (3)
C12B—N1B—C10B—C9B	176.2 (2)	C8A—C9A—C10A—O2A	68.2 (3)
N2B—C9B—C10B—O2B	−179.2 (3)	N2A—C9A—C10A—N1A	7.7 (2)
C5B—C9B—C10B—O2B	60.8 (3)	C5A—C9A—C10A—N1A	128.30 (18)
C8B—C9B—C10B—O2B	−59.4 (3)	C8A—C9A—C10A—N1A	−110.20 (19)
N2B—C9B—C10B—N1B	0.6 (2)	O2A—C10A—N1A—C11A	177.8 (2)
C5B—C9B—C10B—N1B	−119.41 (18)	C9A—C10A—N1A—C11A	−3.8 (2)
C8B—C9B—C10B—N1B	120.4 (2)	O2A—C10A—N1A—C12A	−3.3 (4)
C10B—N1B—C12B—C13B	−82.7 (3)	C9A—C10A—N1A—C12A	175.1 (2)
C11B—N1B—C12B—C13B	92.5 (3)	C10A—N1A—C12A—C13A	106.5 (3)
C10B—N1B—C11B—O3B	−179.9 (2)	C11A—N1A—C12A—C13A	−74.7 (3)
C12B—N1B—C11B—O3B	4.3 (4)	C9A—N2A—C11A—O3A	−173.4 (2)
C10B—N1B—C11B—N2B	−1.4 (3)	C9A—N2A—C11A—N1A	7.8 (2)
C12B—N1B—C11B—N2B	−177.2 (2)	C10A—N1A—C11A—O3A	178.9 (2)
O3B—C11B—N2B—C9B	−179.7 (2)	C12A—N1A—C11A—O3A	−0.0 (3)
N1B—C11B—N2B—C9B	1.9 (3)	C10A—N1A—C11A—N2A	−2.2 (2)
C5B—C9B—N2B—C11B	116.2 (2)	C12A—N1A—C11A—N2A	178.9 (2)
C8B—C9B—N2B—C11B	−116.4 (2)	N2A—C9A—C8A—C7A	174.55 (19)
C10B—C9B—N2B—C11B	−1.5 (2)	C5A—C9A—C8A—C7A	49.0 (2)
C6B—O1B—C7B—C8B	52.1 (3)	C10A—C9A—C8A—C7A	−74.5 (2)
O1B—C7B—C8B—C9B	−63.2 (3)	C9A—C8A—C7A—O1A	−58.6 (3)
N2B—C9B—C8B—C7B	−90.4 (3)	C5A—C6A—O1A—C7A	−7.9 (3)
C5B—C9B—C8B—C7B	38.9 (3)	C1A—C6A—O1A—C7A	173.1 (2)
C10B—C9B—C8B—C7B	159.4 (2)	C8A—C7A—O1A—C6A	37.1 (3)
C1B—C2B—C3B—C4B	−0.7 (4)	C6A—C5A—C4A—C3A	−0.3 (3)
C2B—C3B—C4B—C5B	0.5 (4)	C9A—C5A—C4A—C3A	−179.2 (2)
C6B—C5B—C4B—C3B	0.9 (3)	C1A—C2A—C3A—C4A	0.3 (4)
C9B—C5B—C4B—C3B	−176.3 (2)	C5A—C4A—C3A—C2A	−0.1 (4)

### Hydrogen-bond geometry (Å, º)

Cg is the centroid of ring C1*A*–C6*A*.

**Table:**

*D*—H···*A*	*D*—H	H···*A*	*D*···*A*	*D*—H···*A*
N2*A*—H2*A*···O3*A*^i^	0.86	2.06	2.857 (3)	155
N2*B*—H2*B*1···O3*B*^ii^	0.86	2.44	3.019 (3)	124
N2*B*—H2*B*1···O2*A*^iii^	0.86	2.55	3.290 (3)	145
C1*A*—H1*A*···O3*A*^iv^	0.93	2.45	3.263 (4)	146
C2*B*—H2*B*···O2*A*^v^	0.93	2.58	3.501 (4)	173
C7*B*—H7*B*2···*Cg*^iii^	0.93	2.99	3.680 (3)	129

Symmetry codes: (i) −*x*+2, −*y*+1, −*z*+1; (ii) −*x*+2, −*y*, −*z*; (iii) *x*, *y*−1, *z*; (iv) *x*−1, *y*, *z*; (v) −*x*+1, −*y*+1, −*z*.
