# Supplementary figures and images for: Crystal structure of 1′-ethyl­spiro[chroman-4,4′-imidazolidine]-2′,5′-dione: a hydantoine derivative
Source: Acta Crystallogr E Crystallogr Commun. 2015 Sep 12;71(Pt 10):o705–6. doi: 10.1107/S2056989015016175 (PMC4647411; doi:10.1107/S2056989015016175)

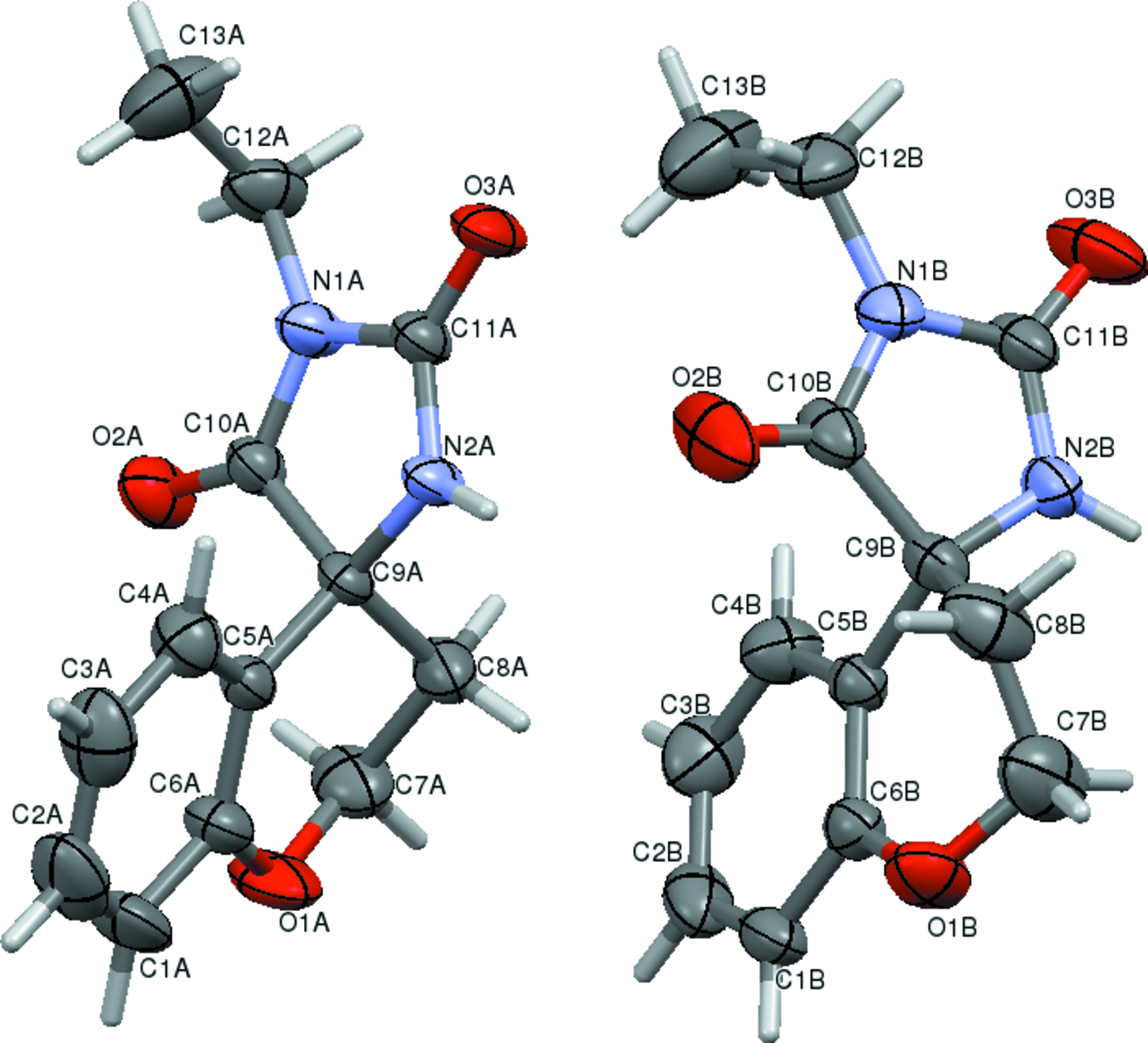

Supplement: Supplementary file 4 [file e-71-0o705-fig1.tif]

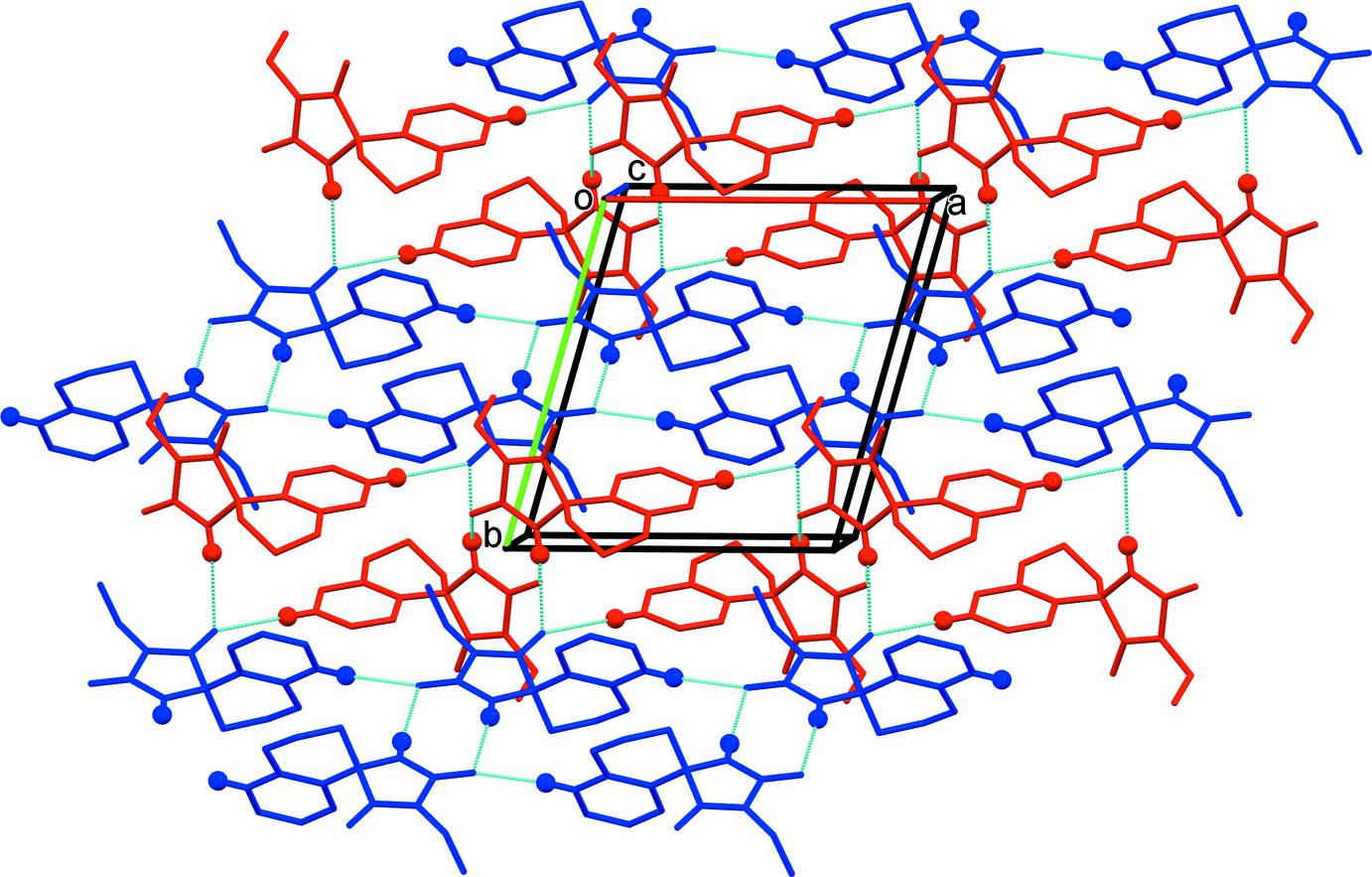

Supplement: Supplementary file 5 [file e-71-0o705-fig2.tif]
